# Authorship: an ethical dilemma of science

**DOI:** 10.1590/S1516-31802005000500008

**Published:** 2005-09-01

**Authors:** Maria Christina Anna Grieger

**Keywords:** Authorship, Ethics, Science, Scientific misconduct, Publications, Autoria, Ética, Ciência, Má conduta científica, Publicações

## Abstract

**CONTEXT AND OBJECTIVE:**

The scientific and technological progress that has taken place since the 1960s has brought an ever-growing volume of scientific research, and inflation in co-authorship. Over this period, it has been observed that an increasing number of publications have listed authors or co-authors whose participation in the published research was minimal or even nonexistent. The objective of this work was to analyze reports in the literature regarding misconduct in authorship: its types, chief causes, consequences and ethical guidelines; and to outline proposals for greater ethical commitment in scientific publication.

**DESIGN AND SETTING:**

Narrative review undertaken at Faculdade de Medicina de Itajubá, Minas Gerais, Brazil.

**METHODS:**

Analysis of publications about authorship using the Medline, Lilacs and SciELO databases.

**RESULTS AND CONCLUSIONS:**

Frequent types of misconduct were gift authorship and divided and redundant publications. The chief causes of these practices seem to be the pressure exerted by academia and the desire for social and professional development. Such factors have brought an increase in unethical behavior. This bias in science continues despite the criteria defined by the International Committee of Medical Journal Editors, the Vancouver group.

**RECOMMENDATIONS:**

Various actions are proposed for educational institutions, research development agencies, regulatory agencies and professional associations. The aim is to establish an evaluation policy that gives primacy to the quality of publications and sets ethical principles for scientific research.

## RESUMO

**CONTEXTO AND OBJETIVO:**

Com o avanço científico e tecnológico ocorrido a partir dos anos 60, surgiu um crescente aumento do número de pesquisas científicas e uma inflação de co-autorias. Ao longo do tempo, observou-se que numerosas publicações mostravam autores ou co-autores cuja participação na pesquisa publicada havia sido mínima ou até mesmo inexistente. O objetivo deste trabalho é analisar, através da literatura, as situações de má-conduta em autoria: tipos, principais causas, conseqüências e normas éticas; e estabelecer propostas para que as publicações científicas apresentem um maior comprometimento ético.

**TIPO DE ESTUDO E LOCAL:**

Revisão narrativa realizada na Faculdade de Medicina de Itajubá, Minas Gerais, Brasil.

**MÉTODO:**

Análise de publicações sobre autoria, através das bases de dados Medline, Lilacs e SciELO.

**RESULTADOS E CONCLUSÕES:**

Freqüentes tipos de má conduta são autoria “presenteada”, fragmentação e duplicação em publicações. As causas que mais induzem a essas situações parecem ser a pressão exercida pela academia e o desejo de ascensão social e profissional. Esse viés na ciência, acrescido de outras formas de autoria antiética, continua até hoje, apesar dos critérios definidos pelo Comitê Internacional de Editores de Periódicos Médicos, o Grupo de Vancouver.

**RECOMENDAÇÕES:**

São propostas várias ações junto às instituições de ensino, agências de fomento à pesquisa, órgãos reguladores e associações de classe, para que se institua uma política de avaliação que priorize a qualidade das publicações, bem como o estabelecimento de preceitos éticos em pesquisa e produção científica.

## INTRODUCTION

In parallel with scientific progress within different fields of human knowledge, there has been a substantial increase in the volume of research. This has been accompanied by an increase in the number of authors in scientific publications.^1–6^ Although such increases have been justified by technological advances that require multidisciplinary and multicenter research projects,^7^ it can be seen that other factors have had a stronger influence on authors’ conduct. One of them may be described as the personal desire for social and professional advancement, tenure, promotion, prestige and fame.^1,2,4,6,8 13^ The other factor, closely linked with the first one and just as important, is the pressure exerted by academia, in which voluminous scientific production is demanded from researchers.^1,3,10–17^ Regrettably, such pressure has encouraged not only increased co-authorship but also other forms of misconduct in authorship.^2^

In 1978, a group of editors assembled in Vancouver, Canada, to create the International Committee of Medical Journal Editors (ICMJE), with the objective of standardizing the publication of scientific articles and standardizing the criteria for writing and authorship. The document they produced set specific authorship criteria, according to which the researcher should fulfill requirements of effective participation in the work in order to be characterized as an author.^18^ Several other committees and organizations have been created since 1990, with the majority of them aimed at developing ethical principles for editors and authors.^19,20^

Despite all the existing rules, there have been no long-lasting changes in conduct regarding the authorship of scientific publications.^3,21-23^ A study conducted in 2002 among medical students revealed that a great proportion of them had adopted unethical practices in their research activities, varying from minor deeds to more grievous acts like falsifying results.^24^ Suggestions for an alternative form of authorship have been put forward: instead of authors, articles would have “contributors”. Such contributors’ participation would be explained in that issue of the publication, so as to assign credit and responsibility to them.^25^ The ICMJE, in a recent update of the rules,^26^ is encouraging editors to adopt this system of a list of collaborators in their journals. Nevertheless, despite all efforts, honorary authors still appear in articles.^5^

Starting from this evidence, some questions arise. What are the causes of the persistence of authorship problems within the scientific environment? Since the rules that were so carefully set up and disseminated by committees and organizations did not have an impact on the problem, what could possibly change the present situation?

With the purpose of analyzing the evolution of the problem of scientific authorship from different angles, a review of literature has been made to search for answers to these questions. Based on these findings, proposals are made for changing the paradigms so that greater ethical commitment may be attained.

## METHOD

The literature review was qualitative, made through bibliographic searches in the Medline (Medical Literature Analysis and Retrieval System Online), Lilacs (Literatura Latino-americana e do Caribe em Ciências da Saúde) and SciELO (Scientific Electronic Library Online) databases. Publications were selected in the form of articles, editorials or letters of content related to numbers of publications, numbers of authors, rules and/or authorship criteria, situations of misconduct in assigning authorship, and recommendations for the solution of the problem. The articles excluded were those exclusively or chiefly concerned with quarrels about authorship in multicenter or cooperative studies, plagiarism, fraud or falsification of data, and conflict of interests.

## RESULTS AND DISCUSSION

### Misconduct in authorship: the subject and its origins

Science strives to unveil the unknown and, for that purpose, research is needed. By bringing forth new achievements, research hopefully improves living conditions on earth.^27^ This is why scientists and researchers are rewarded with outstanding positions in society, achieving prestige, fame and financial support. In their turn, such recognition motivates scientists and researchers, and contributes to the enduring production of science. This quite coherent sequence worked well until the middle of the twentieth century, when the rapid technological advances that mostly took place following the second world war gave rise to a remarkable increase in scientific research.^1,2^ This occurred in several fields of science, but the health sciences had the biggest increase.^2,8^ In one interesting study, Fye identified that the boom in medical publications originated in changes in medical colleges, such as the hiring of full-time professors.^6^

Whatever the yardstick utilized, the truth is that, within universities and research institutes, scientific work multiplied chiefly after the beginning of the 1960s. Funding agencies emerged, requiring the selection of research projects before granting financial support. One of the criteria for evaluating research projects then created was the quantity of papers authored by applicants. Thus arose the famous precept of “publish or perish”, which became widespread throughout academia. According to this, it was absolutely necessary to publish. The dissemination of this idea contributed enormously towards generating an evaluation policy utilized by academic institutions and funding agencies, based on how many papers the researchers had published. This policy had a high potential for creating a wide range of irregular procedures.^1–3,6,12–14,16–18,25,28^ The cycle of “research, publication, promotion, prestige, and more research money” was thus constituted. This cycle would attract those more ambitious professionals and would be responsible for a large number of irrelevant publications.^29^

Obviously, many researchers were not contaminated by the publishing fever. Nevertheless, a significant number of them leaned towards this deviation, in which the author is more important than the work.^30^ Later on, this idea produced another fever: to publish in top journals, thereby making journals more important than the published work.^10^

Following the rising number of publications, authorship inflation came into being. This may be considered the starting point for the phenomenon of undeserved authorship. On the one hand, the strengthening of multidisciplinary teams and multicenter studies has meant larger numbers of co-authors in articles.^7,12,13^ On the other hand, “gift” authorship has arisen. This term characterizes several forms of undue inclusion of authors, for example the inclusion of people in the list of authors with the aim of pleasing somebody and thus receiving something in return.^1,31^ Very often, the reciprocity is the inclusion of the person offering the gift as an author in another publication. In this way, with the dissemination of gift authorship, brilliant careers based on consistent curriculum vitae could be built up without much effort.^1–4,11,17,22,32–35^

This description allows the inference that misconduct in authorship has a multiplicity of causes, such as increasing demand for research, emergence of new research scenarios (multidisciplinary and multicenter), academy pressure due to the quantity-based evaluation model, and the desire for prestige, fame, employment and tenure.

How much each of these factors contributes towards generate unethical conduct has never been determined. Regardless of counter-opinions about the influence of personal promotion,^7^ the majority of the studies consulted for the present review mention this factor as having a strong influence on the subject.^1-2,4,6,8-13,15^ In the introduction of his book “Civilization and its discontents”,^36^ Freud wrote that there is an inescapable impression that people seek success, power and wealth, while underestimating the real values of life. In mentioning career promotion as a cause of authorship inflation, Fye emphatically stated that “ego and economics are the two most powerful stimuli of medical writing”.^6^ In an excellent article about authorship, Lawrence referred to the power of publicity in contemporary society, as a reminder that scientists are not immune to it.^10^

In Brazil the pressure on research exerted by funding agencies has made itself clear in the selective distribution of grants to universities and institutions that have produced larger numbers of publications. This pressure was obviously transmitted onwards to the researchers. More recently, at the end of the 1990s, when the Brazilian Ministry of Education (MEC) established an appraisal system for medical schools and courses, the teaching staff was also evaluated, with a focus on the quantity of its scientific production. The evaluation instrument put into practice for recognizing new medical schools described the evaluation criterion as follows: “… attribution of a grade based chiefly on the number of works published in scientific journals, both national and international, and on authorship of books…” The minimum acceptable rate for articles published in specialized indexed journals is 0.25 per year. Thus, a medical school with 100 teachers should have at least 25 published articles a year in order to obtain grade C. Grade A would only be attained with an index of one article per member of the academic staff per year.^37^ Although this is an understandable way of evaluating performances, the criteria demanded by MEC in its evaluation instrument are liable to criticism since they encourage authorship inflation and other forms of unethical conduct.

### Types of misconduct in authorship and their consequences

In addition to gift authorship, many other types of misconduct are, which may or may not be related to gift authorship: ghost authorship, plagiarism, fragmentation and duplication of works (Table 1).^3^

**Table 1.** Types of authorship misconduct in scientific publications according to Bennett and Taylor^3^

*Gift authorship (Synonyms: guest authorship, honorary authorship, unjustified authorship, undeserved authorship)*	Inclusion, among the authors, of an individual who does not fulfil the requirements for authorship.
*Pressured authorship:*	A person's use of his position of authority in order to be included as an author, regardless of not being thus qualified.
*Ghost authorship (Synonyms: uncompleted authorship, “denial of authorship”)*	Non-inclusion, among the authors, of individuals who played an effective part in the work and were qualified for authorship.
*Fragmentation (Synonyms: separate or divided publication, “salami slicing”)*	Separate publication of various parts of the work, which could have been assembled into one publication.
*Duplication (Redundant publication, “shotgunning”)*	Publication of the same paper in different journals with little or no change at all in its content.

In this literature review, gift authorship is considered to be a frequent type of misconduct in publication. This is authorship granted to people who took part neither in the research work nor in the writing of the paper at any stage.^1–4,11,17,22,32–35,38^ It often occurs with the aim of pleasing somebody so as to obtain some benefit, or as a means of granting some advantage to persons connected with the author (such as relatives or friends, etc.) There is almost always some reciprocity linked to this type of conduct.^17^ But it can also derive from servility or obligation, as is the case when people in charge of institutions, departments, disciplines or services are “gifted” with this type of authorship. Many chairmen impose on subordinates the placing of their names in the list of authors of all the papers published by the department or discipline under their command. This practice is even considered to be a natural occurrence.^1,2,8,11,12,21,31–33,38,39^ Slone regarded gift authorship involving chairmen as a condition of fear or obligation imposed on the researcher.^15^ Whatever the motive for the gift authorship, the winners are: the chairman, who enlarges his production and thus the marks of his academic success; and the true authors, who also enlarge their production and earn the reward for their servility. People who are faced with such competition are the losers. Science also loses out.

Academic promotion was, in a study by Slone, the most often mentioned reason for an honest person to accept undeserved authorship.^15^ Because of this, Leash regarded authorship as “a ‘trading ship in a economic game’ that is bartered for material, information, research subjects, technological expertise, or whatever is required to get the research done”.^9^

Despite being a form of rewarding benefits, gift authorship may result in great harm to those who practice it. There was a case in 1995, in which it was revealed that data had been falsified in two articles published in the British Journal of Obstetrics and Gynaecology.^11,13^ One of the co-authors was the principal researcher's chief and the editor of the journal, which caused enormous repercussion. The explanation given by the person involved in the gift authorship was that “chairmen are always listed among the authors by courtesy”. Another recent affair involving the falsification of data pointed to the existence of this fraud in no less than 25 articles published by one researcher, with a variable list of 20 co-authors who declared themselves innocent, even though a committee nominated to judge the case considered that they were also blameworthy.^40^

With regard to the duplication of scientific works, Kempers pointed out two harmful consequences: grievous distortion of evidence for subsequent meta-analysis studies; and the boosting of the curriculum vitae of those who practice this false scientific production, to the detriment of those who act honestly.^2^ It is important to bear in mind that the emphasis that academic institutions place on publication encourages both duplication and fragmentation of articles. In other words, these forms of wrongdoing are byproducts of the evaluation system for scientific production.^6^ Huth considered that the fragmentation of articles was the commonest and most harmful form of misconduct regarding publications, because of the economic involvement of editors, readers, indexing bodies and libraries.^29^ When fragmentation and duplication of works occur, journals are put in jeopardy, just as much as readers are. Once again, science loses out.

It is important here to comment on another event that followed the increase in research: the proliferation of scientific journals, which is necessary to face the demand, but not always appropriate for methodological and ethical criteria.^1^ Because of this, many scientific journals do not manage to be indexed in major databases such as Medline, which requires high editorial quality. The journals indexed in Medline are thus in high demand from the authors of new studies, who often have to await months until they can see their articles published. On the other hand, such journals have become more selective, through turning down works of good quality or such delays in publication. This expectation creates high anxiety among authors. As a result, such authors push the conclusions from their research and sometimes jeopardize adequate reflection on the results and conclusions.^10^ It is not difficult to see who loses out in this situation.

### Vancouver criteria and other rules

The Vancouver group produced a document entitled “Uniform Requirements for Manuscripts Submitted to Biomedical Journals”,^18^ containing the criteria for assigning authorship in scientific articles. This document was published in several journals and its free publication encouraged by the group. Due to its wide diffusion, a reduction in undeserved authorship was expected. However it did not happen.^2–4,21,33^ Many studies showed that, not only did co-authorship keep on growing, but also there was no impact on other forms of misconduct.^5,21,41^ Because of this, many studies and editors suggested or supported the suggestion that published articles should be accompanied by an explanation of each author's contribution.^3,9,13,25,34^ Rennie suggested that the term “author” be substituted by “contributor”. Thus, for editors and readers, the list of contributions would be the guide to the roles played by each researcher involved in the work. One of the researchers, preferably the one who had had the biggest share in the work, would be responsible for the article as a whole. This person would be named the “guarantor”.^25^

Since originally issued, the criteria defined by the Vancouver group have been updated many times. On the basis of the above suggestions, the 2003 version of the ICMJE criteria included the concept of “contributor” and the proposal that editors should require from authors that they provide a list of the contributions made by everyone participating in any article submitted to their journals, and also that they should identify the person who was responsible for the work as a whole.^26^

In the 2003 version, the criteria for assigning authorship were modified such that credits of authorship must be based on: 1) Substantial contribution to the conception and design or acquisition of data, or analysis and interpretation of data; 2) Drafting the article or revising it critically for important intellectual content; and 3) Final approval of the version to be published. Authors should meet the three conditions.^26^

In addition to ICMJE, several other groups of medical and science editors have founded committees, councils, organizations or associations, mostly between the years 1995 and 2000, directed towards the promotion of ethical and scientific principles.^19,20,42,43^ These groups have tried to act closely with editors and researchers, in seeking to attain adequate procedures for disseminating the results of scientific research and also for managing situations of misconduct. Among the most active groups are the World Association of Medical Editors (WAME) and the Committee on Publication Ethics (COPE). COPE has produced specific rules for editors and authors^19^ and WAME states that one of its objectives is the attainment of the highest level of ethical journalism, with the recognition that, in addition to specific objectives, a medical journal has the social responsibility of improving human conditions and safeguarding science integrity.^20^

In Brazil, the Brazilian Association of Scientific Editors (*Associação Brasileira de Editores Científicos*, ABEC), created in 1985, establishes in its regulations the purpose of bringing together persons and institutions interested in the following: development and improvement of the publication of both technical and scientific journals; improvement of the communication and dissemination of scientific information; and maintenance of the interchange of ideas, discussion of problems and defense of common interests. Among other assignments, ABEC organizes an annual event dedicated to different aspects of scientific publication, including lectures, courses and debates. A code of ethics is being drawn up with the purpose of guiding, disciplining and safeguarding authors and editors.^44^

## FINAL CONSIDERATIONS

### Present situation and prospects for the future

The ICMJE criteria do not seem to have influenced authors’ conduct, even after updated versions in which the adoption of lists of contributions is suggested. In spite of the existence of several other organizations that have also drawn up well-defined criteria that have been published around the world, surveys have shown that no changes in assigning authorship have taken place. That is, various types of misconduct continue to occur.^2–5,21,33,41^ On this basis, and from other evidence, authors like Kempers^2^ and Bennett and Taylor^3^ have proposed that there needs to be a change in the academic evaluation system, and also within the funding agencies,^10^ so that ethical awareness and changes of attitude among researchers may develop.^30^ Fye has suggested an evaluation system in which only the best and most recent works should be submitted.^6^ According to Lock, this idea has been adopted by Harvard University for the evaluation of applicants in its entrance examinations.^11^

It seems possible to envisage that the Vancouver criteria or other similar criteria, together with the requirement that each author's contribution be specified, may in the future encourage a recovery in ethical standards. Nevertheless, starting from the principle that the academic evaluation system favors quantity, to the detriment of quality, and encourages unethical conduct, it seems more plausible and necessary that committees, councils or associations that are created to improve scientific publication, must act intensively among government agencies, evaluators, universities, research institutes and development agencies, with the aim of raising their awareness of the problem that has built up within scientific production. Thus, a list of suggestions and recommendations (Table 2) addressed to these institutions could be the first step towards a real transformation of the present situation. Other institutions like professional associations and societies and regulatory agencies in general should be alerted and advised. Corroborating these ideas, Faintuch suggested a joint effort by medical leaders within their different fields of action, such as journal editors, directors of professional associations, coordinators of medical residence and postgraduate programs, academic authorities and members of ethical committees, so that guidelines can be proposed.^12^ It is worth noting that many professional regulatory councils in the field of healthcare in Brazil do not include research authorship within their codes of ethics. Nutrition, physiotherapy, physical education and psychology are examples. The codes of ethics for medicine, nursing, pharmacy and dentistry have specific articles that govern the matter of authorship in scientific publications (articles 137, 56, 18 and 34 of these codes, respectively).^45–48^

**Table 2.** Recommendations for authorship conduct patterns according to various institutions

Recommendations	To whom addressed
The author should use the Vancouver criteria for assigning authorship in scientific works to be published.	Scientific journals, publishing companies, universities and research institutes.
Research to be published or presented in events must exhibit written declarations regarding the contribution and responsibility of each author.	Scientific journals, publishing companies, education and research institutes, and professional societies.
Research ethics committees should require that the authors of research projects should provide descriptions of the activities envisaged for each author.	National research ethics council and research ethics committees.
Ethical rules for research activities should be included in professional codes of ethics.	Professional regulatory agencies (regional and federal councils).
The ethics of research activities should be included within the content of universities courses.	Ministry of Education, universities and educational associations.*
An evaluation policy for higher education institutions that emphasizes the quality of scientific production instead of stimulating quantity should be adopted.	Ministry of Education and research stimulation agencies.†
The need to transform the present situation regarding scientific publication into an acceptable ethical condition should be disseminated within the academic environment.	Universities, regional and federal councils,‡ and educational associations, etc.

* In Brazil: ABEM (Associação Brasileira de Educação Médica);

† In Brazil: CAPES (Coordenação de Aperfeiçoamento de Pessoal de Nível Superior) and CNPq (Conselho Nacional de Desenvolvimento Científico e Tecnológico);

‡ In Brazil: CRM (Conselho Regional de Medicina) and CFM (Conselho Federal de Medicina).

It is important that the proposed recommendations be adopted by these institutions so that a paradigm shift may occur. The recommendations are expected to contribute strongly towards the establishment of an evaluation policy that gives primacy to the quality of the publication, along with the ethical commitment of the researchers.
